# Finger Muscle Attachments for an OpenSim Upper-Extremity Model

**DOI:** 10.1371/journal.pone.0121712

**Published:** 2015-04-08

**Authors:** Jong Hwa Lee, Deanna S. Asakawa, Jack T. Dennerlein, Devin L. Jindrich

**Affiliations:** 1 Department of Mechanical and Aerospace Engineering, Arizona State University, Tempe, Arizona, United States of America; 2 Department of Kinesiology, California State University, San Marcos, California, United States of America; 3 Department of Physical Therapy, Movement, and Rehabilitation Sciences, Bouvé College of Health Sciences, Northeastern University, Boston, Massachusetts, United States of America; University of Utah, UNITED STATES

## Abstract

We determined muscle attachment points for the index, middle, ring and little fingers in an OpenSim upper-extremity model. Attachment points were selected to match both experimentally measured locations and mechanical function (moment arms). Although experimental measurements of finger muscle attachments have been made, models differ from specimens in many respects such as bone segment ratio, joint kinematics and coordinate system. Likewise, moment arms are not available for all intrinsic finger muscles. Therefore, it was necessary to scale and translate muscle attachments from one experimental or model environment to another while preserving mechanical function. We used a two-step process. First, we estimated muscle function by calculating moment arms for all intrinsic and extrinsic muscles using the partial velocity method. Second, optimization using Simulated Annealing and Hooke-Jeeves algorithms found muscle-tendon paths that minimized root mean square (RMS) differences between experimental and modeled moment arms. The partial velocity method resulted in variance accounted for (VAF) between measured and calculated moment arms of 75.5% on average (range from 48.5% to 99.5%) for intrinsic and extrinsic index finger muscles where measured data were available. RMS error between experimental and optimized values was within one standard deviation (S.D) of measured moment arm (mean RMS error = 1.5 mm < measured S.D = 2.5 mm). Validation of both steps of the technique allowed for estimation of muscle attachment points for muscles whose moment arms have not been measured. Differences between modeled and experimentally measured muscle attachments, averaged over all finger joints, were less than 4.9 mm (within 7.1% of the average length of the muscle-tendon paths). The resulting non-proprietary musculoskeletal model of the human fingers could be useful for many applications, including better understanding of complex multi-touch and gestural movements.

## Introduction

Dexterous manipulation often involves complex movements of several fingers. For example, grasping or pinching can involve the coordinated activations of many hand muscles [1]. Movements similar to pinching and grasping are commonly used as inputs for human-computer interfaces (HCIs) such as a smart phone and tablet computer touch screen. The “multitouch” interfaces of these devices often require complex, multi-finger gestures or gesture sequences on the touch screen [2]. However, we have little understanding of whether the cumulative effects of long-term exposures can lead to injuries such as musculoskeletal disorders (MSDs). One way to identify exposure to forces that may lead to injury is to estimate muscle and tendon forces during repetitive activities. However, individual muscle tension or stress is difficult to measure *in vivo* [3,4].

Musculoskeletal models can be helpful for understanding many aspects of biomechanics and motor control. Biomechanical models can help to understand hand anatomy [5,6]. Models have also contributed to understanding upper-extremity biomechanics, including internal muscle loading during motor tasks [7–10]. Musculoskeletal models have also helped to understand neural control of hand and arm movements. For example, models helped to identify the function of the intrinsic hand [11,12] and thumb and index finger muscles [13,14], and to study movement coordination of the interphalangeal joints [15,16].

However, existing models have several limitations. First, many models are developed with proprietary or commercial software, and are not generally available for use. Second, many models are two-dimensional (2D), whereas movements are often in three-dimensional (3D) space. Third, many models focus on specific joints or sets of joints and do not include the entire upper extremity. Finally, even models that incorporate all of the major skeletal segments of the hand and arm do not currently include all of the muscles potentially responsible for actuating complex finger motions.

Muscle attachment points have been experimentally measured for both intrinsic and extrinsic muscles of the hand [5]. However, published attachments cannot be directly used in the OpenSim model [9]. The 50th percentile male used for the OpenSim model differs from the specimens used for experimental measurements. For example, the OpenSim model and experimental specimens have different sized segments, segment proportions, joint center positions, rotational axis orientations and anatomical coordinate systems. Therefore, directly adding experimentally measured muscle attachments to the OpenSim model results in different modeled moment arms than those experimentally measured for the same muscles. Muscle moment arm is an important functional measure because it determines the joint torques that result from muscle forces. Therefore, to create a musculoskeletal model capable of evaluating muscle function, it is necessary to determine sets of muscle attachments that result in moment arms that are representative of human subjects.

Several studies have measured moment arm *in vivo* [17–19] and *in situ* [20–24]. However, to our knowledge, moment arms for all muscles of the middle, ring and little fingers have not been reported. Moreover, even when moment arms are known, the specific muscle attachments are unknown and indeterminate: many potential muscle attachments can result in similar moment arms [25].

To overcome some of these limitations, we seek to add intrinsic and extrinsic finger muscles to a non-proprietary, 3D musculoskeletal model of the upper extremity [9,26]. The OpenSim upper-extremity model currently incorporates muscles at the shoulder and elbow. Adding finger muscles is a necessary component of using the model to better understand dexterous finger movements. The OpenSim model is particularly suited to help understand the role of segment interactions and the consequences of multi-joint articulation of finger muscles.

Therefore, we sought to identify muscle attachments for the intrinsic and extrinsic finger muscles within the OpenSim upper-extremity model that resulted in the functionally important characteristic of matching experimentally measured moment arms.

The procedure we used to identify muscle attachments involved two steps. First, we determined muscle moment arms from experimentally measured tendon locations [5]. We used the “partial velocity” method calculation [27,28] to reproduce anatomical moment arms from experimentally measured muscle attachment points [5]. Successful prediction was considered to be calculated moment arms within one standard deviation (S.D) of experimental measurements (S.D = 2.5 mm; [6,20]). Second, given a moment arm curve, we identified muscle-tendon paths using a data-driven optimization that is capable of determining muscle attachments whose resulting moment arms matched experimental measurements [25]. We considered successful approximation to be optimized moment arms within 10% of experimentally derived values [29,30].

## Methods

We expanded an existing musculoskeletal model of upper extremity on the OpenSim platform to include finger muscles (2.3.2, Simbios, Stanford, CA; [9,31]). Currently, the shoulder, elbow, forearm, wrist, thumb and index finger are modeled with 15 joint degrees of freedom (DOFs) and 50 muscle elements. The existing model is actuated by 50 muscle compartments including extrinsic muscles of the thumb and index finger. The rotational axes and centers for the thumb joints are based on measured values [32,33], and those for the index finger joints are modeled as the long axis of cylinders fit to the articular surfaces of the metacarpal and phalangeal bones.

### Musculoskeletal Model

We added custom joints to the middle, ring and little fingers of the OpenSim model because only the thumb and index finger currently have active joints [9]. Each finger was modeled to have four DOFs linking four successive bones: metacarpal bone, proximal phalange, middle phalange and distal phalange. These four bone segments were linked with three joints: metacarpophalangeal (MCP), proximal interphalangeal (PIP) and distal interphalangeal (DIP). The MCP joint was capable of flex/extension and ab/adduction as a universal joint (2 DOFs). The PIP and DIP joints were limited to flex/extension as revolute joints (1 DOF). We constrained joint angles to be within their physiological range of motion (RoM) from experimental measurements [20,23,34]. The MCP joints were modeled with a RoM of 0° (extension:-) to 90° (flexion: +) as well as 0° (adduction:-) to 30° (abduction: +). The PIP and DIP joints were also modeled with a RoM of 0° to 90° (flexion: +) and 50° (flexion: +), respectively.

For all fingers, we modeled the following muscles: *terminal extensor (TE)*, *extensor slip (ES)*, *radial band (RB)*, *ulnar band (UB)*, *dorsal interosseous or radial interosseous (RI)*, *lumbricals (LU)*, *palmar interosseous or ulnar interosseous (UI)*, *flexor digitorum profundus (FDP)*, *flexor digitorum superficialis (FDS) and extensor digitorum communis or long extensor (EDC)*. The wrapping surfaces for all fingers were based on previous studies [9,25,31,35]. We modified the wrapping surface parameters (e.g., dimension, orientation and position) to prevent the muscles passing into the bone for the entire range of movements.

### Scaling Model and Moment Arms

To normalize for differences among data sets and reproduce hand skin surface from the bony segments in the OpenSim model, we assumed that all linear dimensions scaled isometrically, as found for the ratio among the length of phalanx, the width and thickness of each joint [5,25,36–39]. We scaled measured anthropometric data to the OpenSim model dimensions to describe muscle-tendon paths within OpenSim (Table 1), then normalized moment arms to the length of the middle phalanx [5,25].

**Table 1:** Anthropometric index finger dimensions of cadaveric specimens [20] and OpenSim model (mm).

	**Specimen bony dimensions**	**OpenSim bony dimensions**	**Skin surface scaled**
**Distal phalanx length**	19.67±1.03	19.10(Δ0.57)	30.65
**Middle phalanx length**	24.67±0.98	25.10(Δ0.43)	27.22
**Proximal phalanx length**	43.57±0.98	42.60(Δ0.97)	50.86
**DIP joint thickness**	5.58±0.92	4.95(Δ0.63)	14.38
**PIP joint thickness**	7.57±0.45	7.31(Δ0.26)	18.86
**MCP joint thickness**	15.57±0.84	17.08(Δ1.51)	27.80

Symbol (±) indicates standard deviation in interspecimen variation. Lengths of the phalanges in OpenSim model are calculated by the distance between the origins of two coordinate systems in three-dimensional (3D) Cartesian space, e.g., the center of rotation at MCP and the center of rotation at PIP. Parentheses (Δ) in OpenSim bony dimensions express difference between model dimension and specimen dimension. Skin surface set is scaled in three-dimensions to preserve measured anatomical proportions [36–39]. These skin surface (external dimensions) function as upper boundary constraints during optimization.

### Reproducing Moment Arms from Experimentally Measured Attachments

Because experimental moment arm curves are available only for the index finger at the MCP joint, it was necessary to calculate moment arm values from measured muscle attachment points for the middle, ring and little fingers. We calculated moment arms from tendon attachment locations [5] using the “partial velocity” method [28]. The partial velocity method provides a consistent technique to compute the moment arms of muscles crossing many types of joints [27].

To validate the moment arms calculated using the partial velocity method, we compared moment arms calculated from muscle attachments for the index finger [5] to measured moment arms [20]. Experimentally measured moment arms as a function of joint angle were only available for the MCP joint of the index finger, with the exception of the FDS muscle [40]. Therefore, we limited comparisons of moment arm curves to the index finger MCP joint and the FDS muscle for all of the fingers.

### Muscle Attachment Determination

We used a data-driven optimization approach to identify muscle attachment sites that resulted in moment arms that most closely matched the experimentally derived curves over the joint range of motion. We performed optimizations using the same methods that were used to find attachment locations of the index finger [25]. The optimization consists of three parts: 1) an objective function, 2) boundary conditions and 3) an inequality constraint. We defined the objective function as the root mean square (RMS) error between the experimentally derived moment arms, ${\text{r}}_{\text{j}}\left({\vec{\text{q}}}_{\text{i}}\right)$ and the model-estimated moment arms, ${\hat{\text{r}}}_{\text{j}}\left({\vec{\text{q}}}_{\text{i}},\vec{\text{x}}\right)$. The optimization searched for the minimum values of RMS error ($f\left(\vec{x}\right)$) over the domain of attachment points ($\vec{x}$) and joint angles ($\vec{q}$) that satisfy both flex/extension and ab/adduction moment arm relationships (g_j_ (x)). Moment arm curves are different in magnitude and shape at every joint. However, we do not currently have criteria to weight errors at different joints. Therefore, we summed RMS errors for each muscle over all joints in the objective function.

$$\begin{array}{l} \text{Minimize }f\left(\vec{x}\right)=\sqrt{\overset{m}{\underset{i=1}{\sum }}\frac{{\left[r_{j}\left({\vec{q}}_{i}\right)-{\hat{r}}_{j}\left({\vec{q}}_{i},\vec{x}\right)\right]}^{2}}{m}}\\ \text{Subject to }lb_{j}\leq x_{j}\leq ub_{j}\\ {\text{ g}}_{\text{j}}\left(\vec{x}\right)-\varepsilon_{\text{j}}\leq 0 \end{array}$$

We defined the optimization parameters and variables as:

$\vec{x}$ Muscle attachment points (distal and proximal; $\vec{x}\in R^{6}$)

${\vec{q}}_{i}$ Joint angle (resolution (*i*) of 100 increments (*m*) covering the RoM)

$r_{j}\left({\vec{q}}_{i}\right)$ Experimentally measured moment arms

${\hat{r}}_{j}\left({\vec{q}}_{i},\vec{\text{x}}\right)$Model-estimated moment arms

*i* Joint motions (flex/extension or ab/adduction)

*j* Individual muscles (*j* = 40)

*lb*_*j*_ Lower bound (bone surface)

*ub*_*j*_ Upper bound (skin surface)

ε_j_ Maximum standard deviation of experimental moment arms

${\text{g}}_{\text{j}}\left(\vec{x}\right)$ RMS error of ab/adduction moment arms during flexion/extension movements

Second, the boundary conditions constrained muscle attachments to be between the bone (the lower bound, *lb*_*j*_ [9]) and the skin surface (the upper bound, *ub*_*j*_ [36,38,39]).

Third, we imposed an inequality constraint on ab/adduction ($g_{j}\left(\vec{x}\right)\leq \varepsilon_{\text{j}}$). Muscle-tendon paths have moment arms for both flex/extension and ab/adduction at the MCP joint. However, measured moment arms for ab/adduction are more uncertain than those for flex/extension. For example, ab/adduction moment arms have higher standard deviations than flex/extension moment arms, reflecting either measurement or anatomical variability [5,20,21,23]. Moreover, ab/adduction moment arm values depend on joint angles of the MCP, PIP and DIP joints [41]. However, the specific postures used to measure ab/adduction moment arm values are not available. Therefore, we included ab/adduction in the optimization by using an inequality constraint $\left(g_{j}\left(\vec{x}\right)\leq \varepsilon_{\text{j}}\right)$ to find solutions where muscle attachments $\left(\vec{x}\right)$ resulted in moment arms within the standard deviations about experimentally measured moment arms, ($g_{j}\left(\vec{x}\right)\leq \varepsilon_{\text{j}}$). The standard deviations for ab/adduction, ε_j_, were 2.5 mm for extrinsic muscles and 1.7 mm for intrinsic muscles [20]. Our optimization was implemented using Simulated Annealing [42] and Hooke-Jeeves [43] algorithms. Both algorithms were chosen because they have been used successfully for musculoskeletal modeling [25].

Initial parameter selection can influence optimization in complex search spaces [13,25,43]. Therefore, we performed sensitivity analyses for all attachments (${\vec{x}}_{j}$). We performed optimizations using 26 different starting points (${\vec{x}}_{0}$). The starting points included experimental estimates attachment locations [5] and where possible existing OpenSim model attachments [9]. Optimizations from different initial conditions can discover multiple solutions, which require a criterion to select a single set of attachment points [25]. We assumed that the optimized attachment points that were nearest to the experimentally measured locations and resulted in smooth tendon paths were the most anatomically reasonable.

To determine the locations of experimentally measured attachments in the OpenSim coordinate systems, we transformed attachments from the An et al. (1979) reference frames to OpenSim. We constructed a transformation matrix that mapped translation and rotation in three dimensional (3D) Cartesian coordinates (3D Euclidean space). We approximated kinematic parameters: segment rotation (θ), segment distance (d), joint twist (α) and joint offset (a) from the OpenSim model [9]. X coordinate axes were assigned to the joint axes (-: ulnar to +: radial deviation), Y coordinate axes were assigned to the phalangeal bone (-: distal to +: proximal phalanx), and Z coordinate axes were determined by the right hand rule (-: palmar to +: dorsal direction) in the OpenSim coordinate system. Primary coordinate systems of An et al. (1979), located at the approximate center of rotation of the distal, proximal phalangeal and metacarpal heads (2, 4 and 6 reference frames), were translated to OpenSim PIP, MCP joints and middle of metacarpal bone coordinate systems, respectively. Secondary coordinate systems of An et al. (1979), a translation of the proximal systems to the centers of the concave articular surface (1, 3 and 5 reference frames) were translated to OpenSim DIP, PIP and MCP joint coordinate systems, respectively.

Attachment points closest to experimentally measured values were chosen for analysis and presentation. If several candidate attachment points had Euclidean distances to the experimentally measured point within the standard deviation of experimental measurements (28% of middle phalanx length [5]), we chose the path with the largest radius of curvature (the “smoothest” path). Curvature is the rate of change of tendon path direction (i.e. deviation of the tendon path from a straight line). The radius of curvature was determined from three successive attachments, e.g., origin, via and insertion points [25]. The tendon path with the largest radius of curvature was considered the smoothest path and chosen for analysis and presentation. To measure the agreement between model-optimized and experiment-measured moment arm curves, we estimated the variance accounted for (VAF) using the equation:

VAF=100×[1−∑(r→−r→^)2∑r→2]

We implemented optimizations in Matlab (2012a, Mathworks, Natick, MA) using the OpenSim Application Programming Interface (API; OpenSim 2.0 Doxygen) to compute moment arms.

## Results

### Moment Arms Calculated from Muscle Attachments Fitted Experimentally Measured Values for the Index Finger

**Fig 1:**
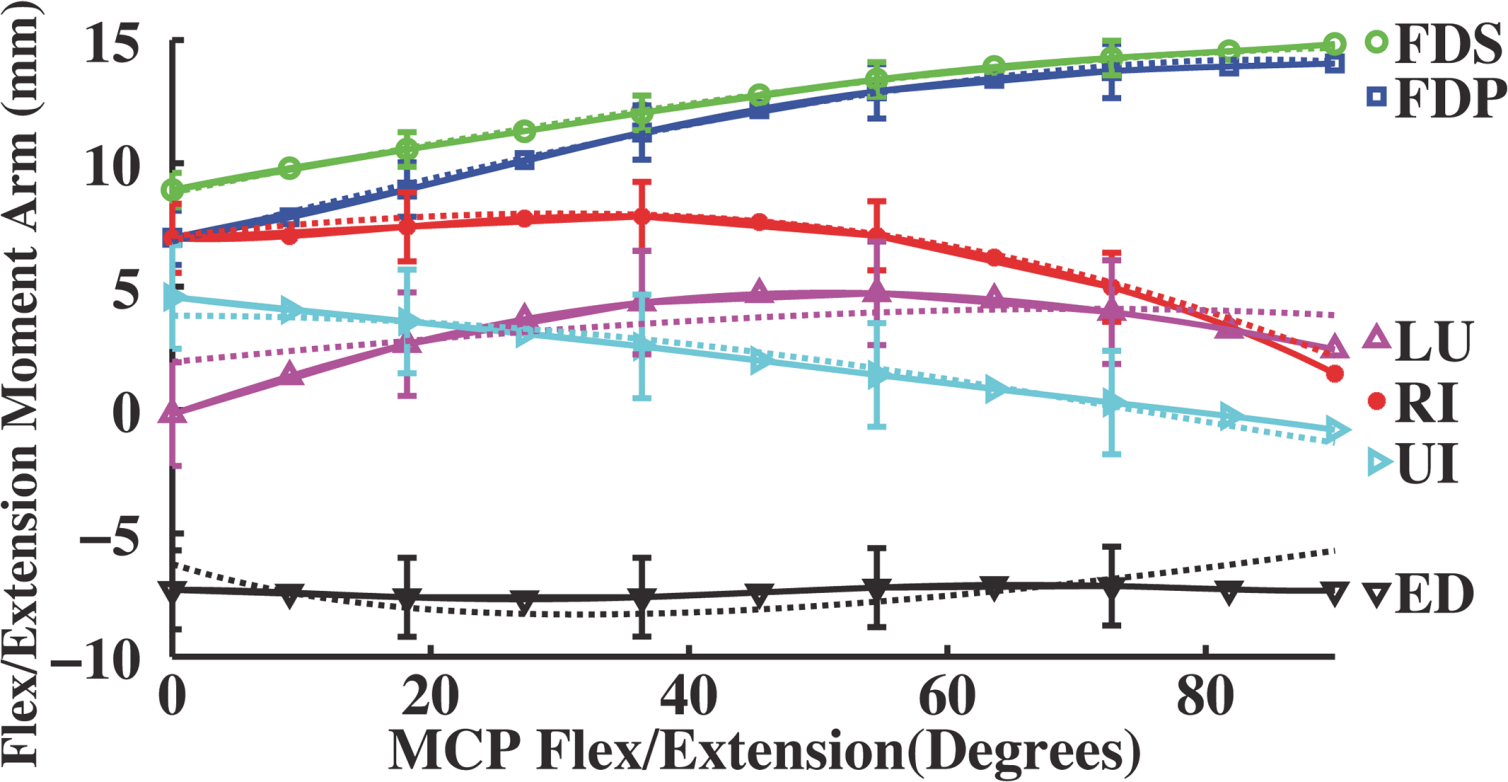
Measured and derived flex/extension moment arms (mm) as a function of flexion (+)/ extension (-) at the MCP joint of the index finger. Dotted moment arm values are derived from experimental muscle attachments [5], and solid moment arm values are direct measurements (n = 7 specimens with mean and standard deviation (error bar); [20]). Positive values indicate flexion moment arms, negative values indicate extension moment arms, and 0° is full extension.

Moment arms calculated using the partial velocity method fitted those measured at the MCP joint of the index finger (Fig 1; Table 2). Over the entire range of motion, calculated moment arms derived from anatomical muscle attachments [5] were within one standard deviation of experimentally measured moment arms [20]. Variance accounted for (VAF) averaged 75.5% across all index finger muscles, ranging from minimum (min.) 48.2% (UI) to maximum (max.) 99.5% (FDS). VAF for the UI muscle was low because of the small value for the moment arms of this muscle. However, RMS error for UI muscle was within one standard deviation (S.D) of experimentally measured moment arms (RMS error = 2.0 mm < measured S.D = 2.1 mm), implying that the calculated moment arm was reasonable. VAF were 96.5% for extrinsic tendons and 86.9% for intrinsic muscles (flex/extension).

**Table 2:** Derived mean moment arms (MA) from experimentally measured muscle attachments [5].

**Joint**	**RoM**		**FDP**	**FDS**	**EDC**	**LU**	**RI**	**UI**
**MCP2**		**MA**	11.5	12.4	-7.4	3.5	6.6	2.0
	**0°~90°**	**(VAF)**	(98.7%)	(99.5%)	(91.0%)	(77.0%)	(95.9%)	(87.9%)
	**Flex/Ext**	**RMS**	Δ0.2	Δ0.1	Δ0.7	Δ0.8	Δ0.3	Δ0.3
		**(Error)**	(1.2%)	(0.5%)	(8.0%)	(23.4%)	(4.3%)	(73.9%)
		**MA**	-2.8	2.2	2.5	4.3	4.1	-6.8
	**0°~30°**	**(VAF)**	(68.2%)	(63.0%)	(53.2%)	(68.1%)	(56.8%)	(48.2%)
	**Ab/Add**	**RMS**	Δ0.8	Δ3.4	Δ2.6	Δ1.5	Δ3.1	Δ2.0
		**(Error)**	(31.2%)	(41.6%)	(99.4%)	(10.7%)	(79.6%)	(43.0%)

All are expressed in millimeters (mm) with flexion (+)/ extension (-) and abduction (+)/ adduction (-) at the MCP joint (degree) of the index finger. (VAF) represents Variance Accounted For, RMS represents root mean square error (Δ) between derived moment arms [5] and experimentally measured values [20], and (Error) represents percentage error, respectively.

### Moment Arms Calculated from Muscle Attachments were Reasonable for All Finger Muscles

**Fig 2:**
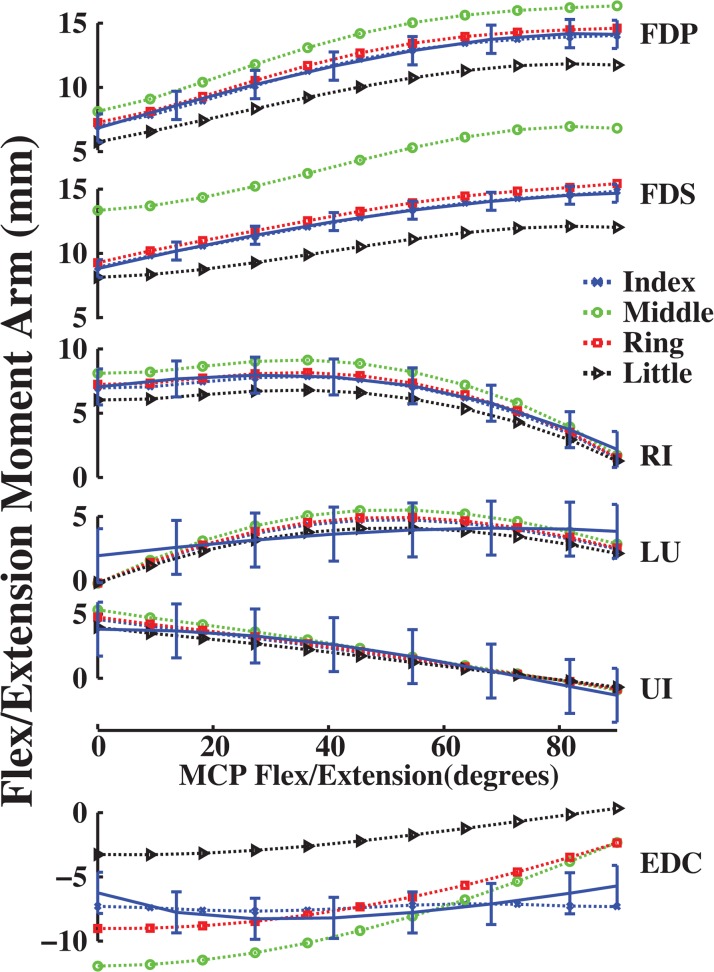
Flex/extension moment arms (mm) as a function of flexion (+)/ extension (-) at the MCP joint of the all fingers. Dotted moment arm values are derived from experimentally measured muscle attachments [5], and solid moment arm values are direct measurements (n = 7 specimens with mean and standard deviation; [20]). Positive values indicate flexion moment arms, negative values indicate extension moment arms, and 0° is full extension. Blue, green, red and cyan colors represent index, middle, ring and little finger moment arms, respectively.

**Fig 3:**
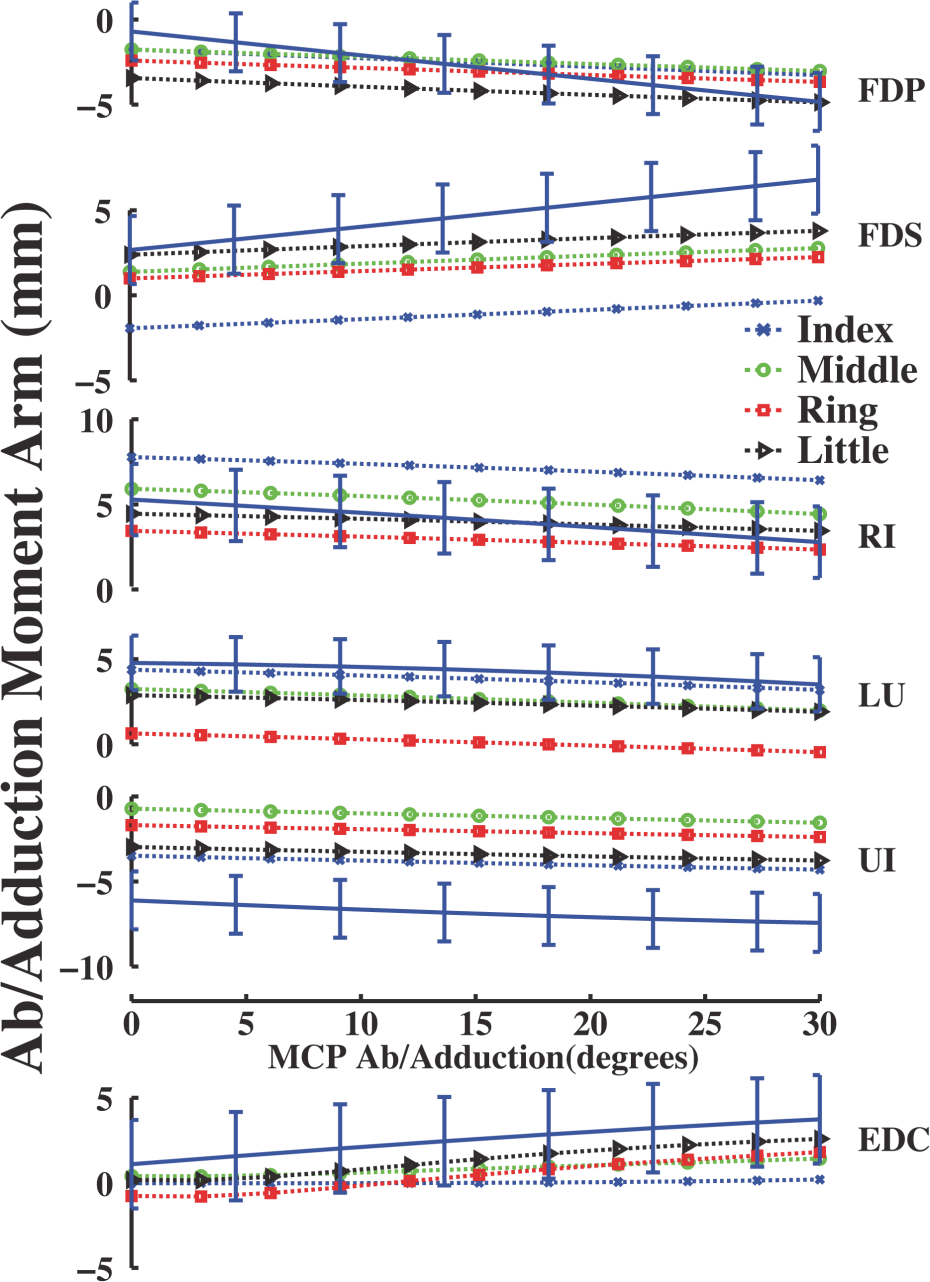
Ab/adduction moment arms (mm) as a function of abduction (+)/ adduction (-) at the MCP joint of the all fingers. Symbols are the same as in Fig 2.

**Fig 4:**
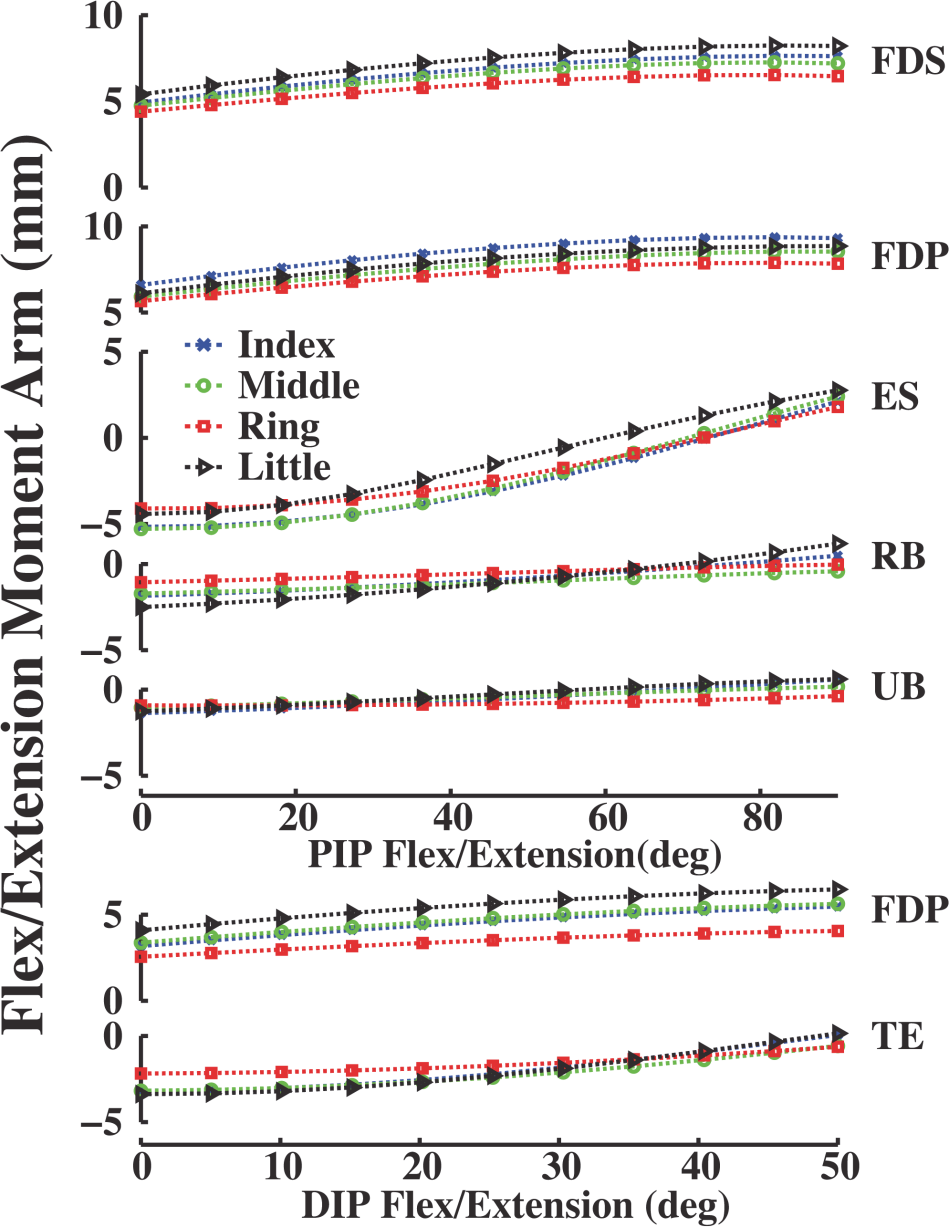
Flex/extension moment arms (mm) as a function of flexion (+)/ extension (-) at the PIP and DIP joints of the all fingers. Symbols are the same as in Fig 2.

Calculated moment arms for the middle, ring, and little finger muscles were reasonable based on moment arm magnitude and variation with joint angle. Specifically, all calculated flex/extension moment arms had an ordering of magnitude that was comparable to the ordering of finger dimensions. Moment arms for the middle finger were largest, ring or index second and little smallest (Figs 2–4). Moreover, calculated moment arms were consistent functions of joint angle. Calculated moment arms of FDP, FDS, RI, UI and LU increased with finger flexion, while those of TE, ES and EDC decreased with flexion for the index, middle, ring and little fingers. Moment arm variation with flex/extension was consistent across all finger joints.

Unlike flex/extension moment arms, ab/adduction moment arms did not show ordering that paralleled finger dimensions. For example, calculated FDS and EDC ab/adduction moment arms for the little finger were larger than those for the middle finger (Fig 3). However, little finger moment arms were consistent with FDS and EDC muscle locations, which were longer than measured middle finger muscle locations from the MCP joint (Euclidean distance, *d*_*little*_ = 0.52 > *d*_*middle*_ = 0.40 × middle phalanx length). Therefore, the partial velocity calculation predicted moment arm curves consistent with expectations based on finger anatomy.

### Optimized Moment Arms Matched Calculated Values Derived from Anatomical Attachments

**Fig 5:**
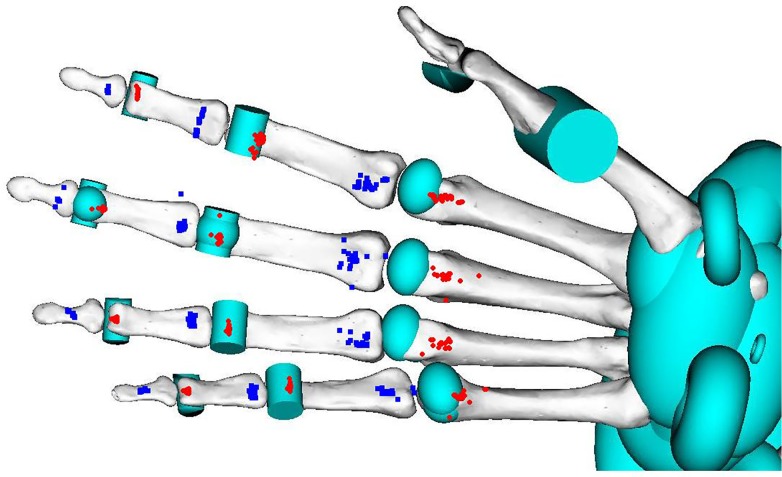
Muscle attachment points for modeled FDP muscles at the MCP, PIP and DIP joints. Red circles and blue squares denote proximal and distal points, respectively.

Optimization identified multiple muscle attachments that locally minimized RMS error (Fig 5). The average Euclidean distance from experimentally measured attachment points to the closest, most smooth optimized muscle attachments (i.e., those selected for analysis and presentation) was 4.9 mm, or 7.1% of the average length along the muscle-tendon pathways.

**Fig 6:**
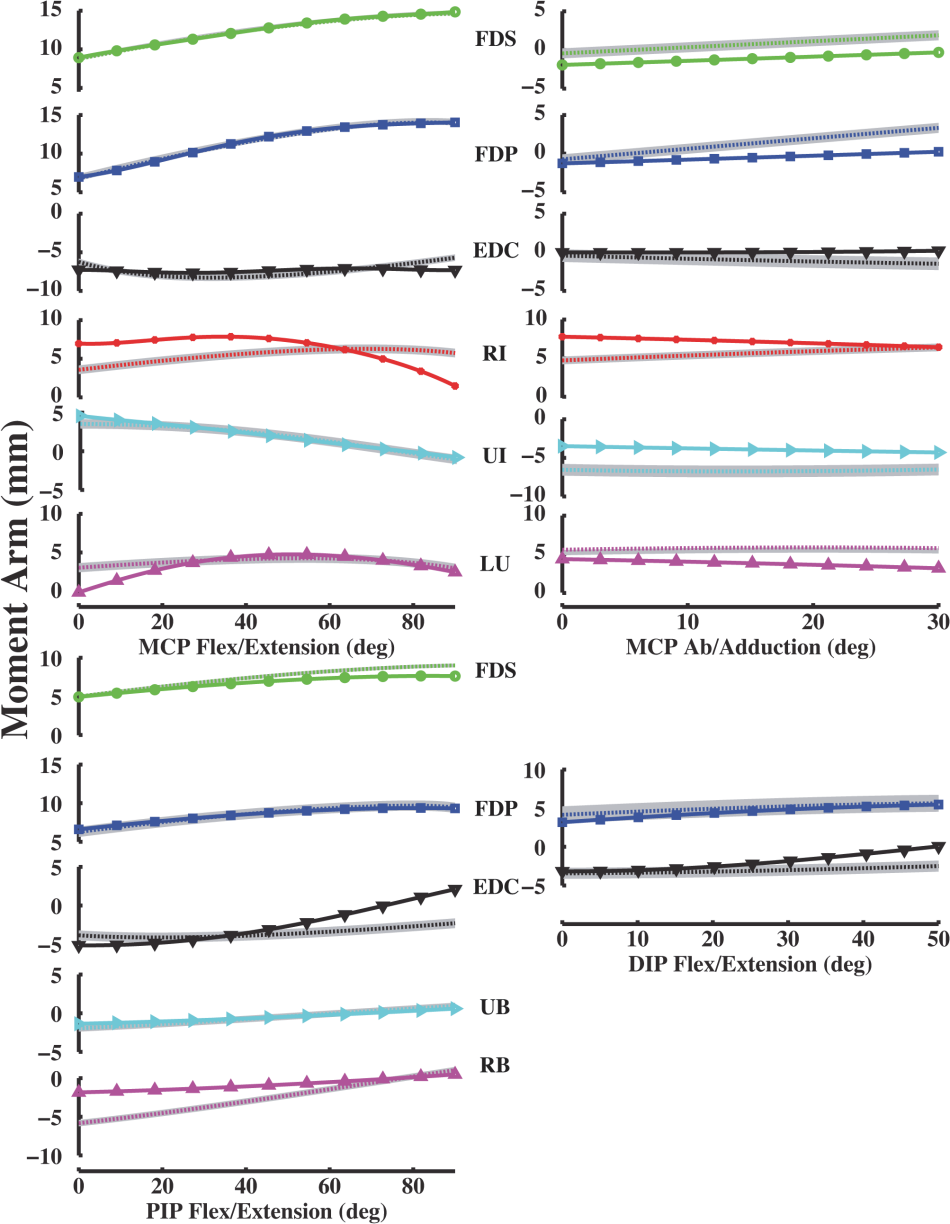
Index finger moment arm values (mm). Left MCP curves, PIP and DIP curves represent flex/extension moment arms as a function of flexion (+)/ extension (-). Right MCP curves represent ab/adduction moment arms as a function of abduction (+)/ adduction (-). Solid curves (with plot markers) represent experimentally derived moment arms from anatomical attachment locations, and dotted curves represent optimally estimated moment arms from data-driven optimizations. Positive values indicate flexion moment arms, negative values indicate extension moment arms, and 0° flexion is full extension, respectively.

**Fig 7:**
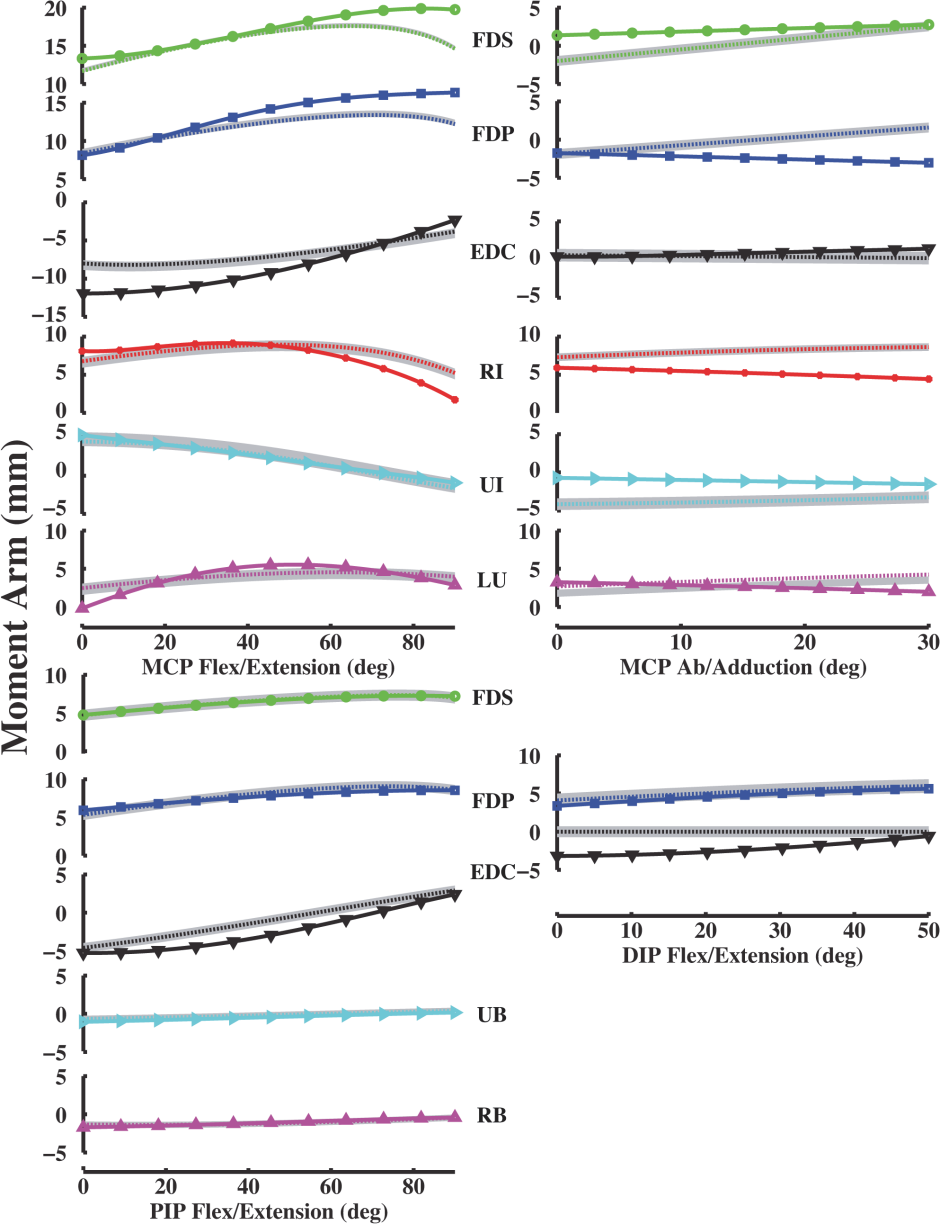
Middle finger moment arm values (mm). Left MCP curves, PIP and DIP curves represent flex/extension moment arms as a function of flexion (+)/ extension (-). Right MCP curves represent ab/adduction moment arms as a function of abduction (+)/ adduction (-). Solid curves (with plot markers) represent experimentally derived moment arms from anatomical attachment locations, and dotted curves represent optimally estimated moment arms from data-driven optimizations.

**Fig 8:**
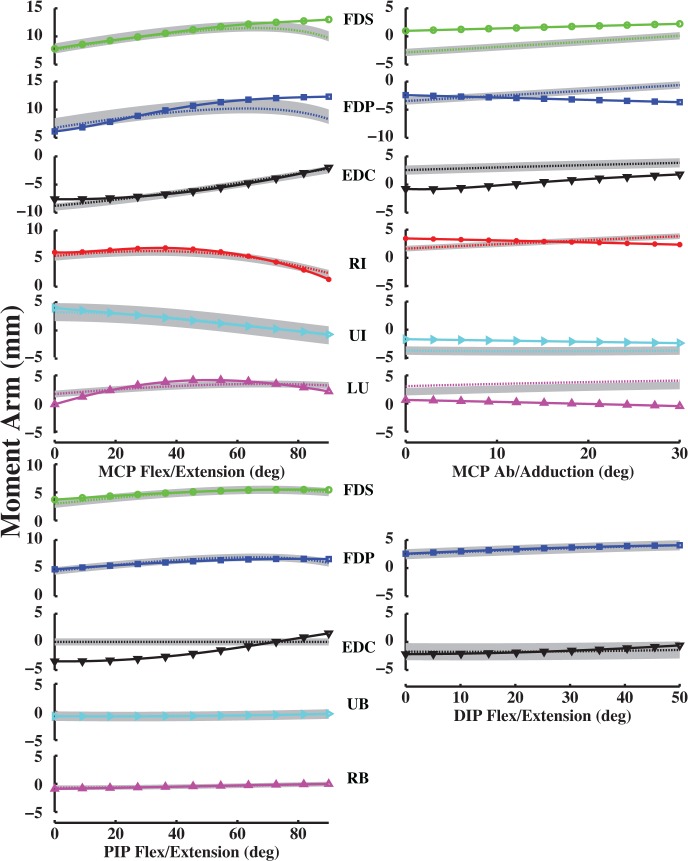
Ring finger moment arm values (mm). Left MCP curves, PIP and DIP curves represent flex/extension moment arms as a function of flexion (+)/ extension (-). Right MCP curves represent ab/adduction moment arms as a function of abduction (+)/ adduction (-). Solid curves (with plot markers) represent experimentally derived moment arms from anatomical attachment locations, and dotted curves represent optimally estimated moment arms from data-driven optimizations.

**Fig 9:**
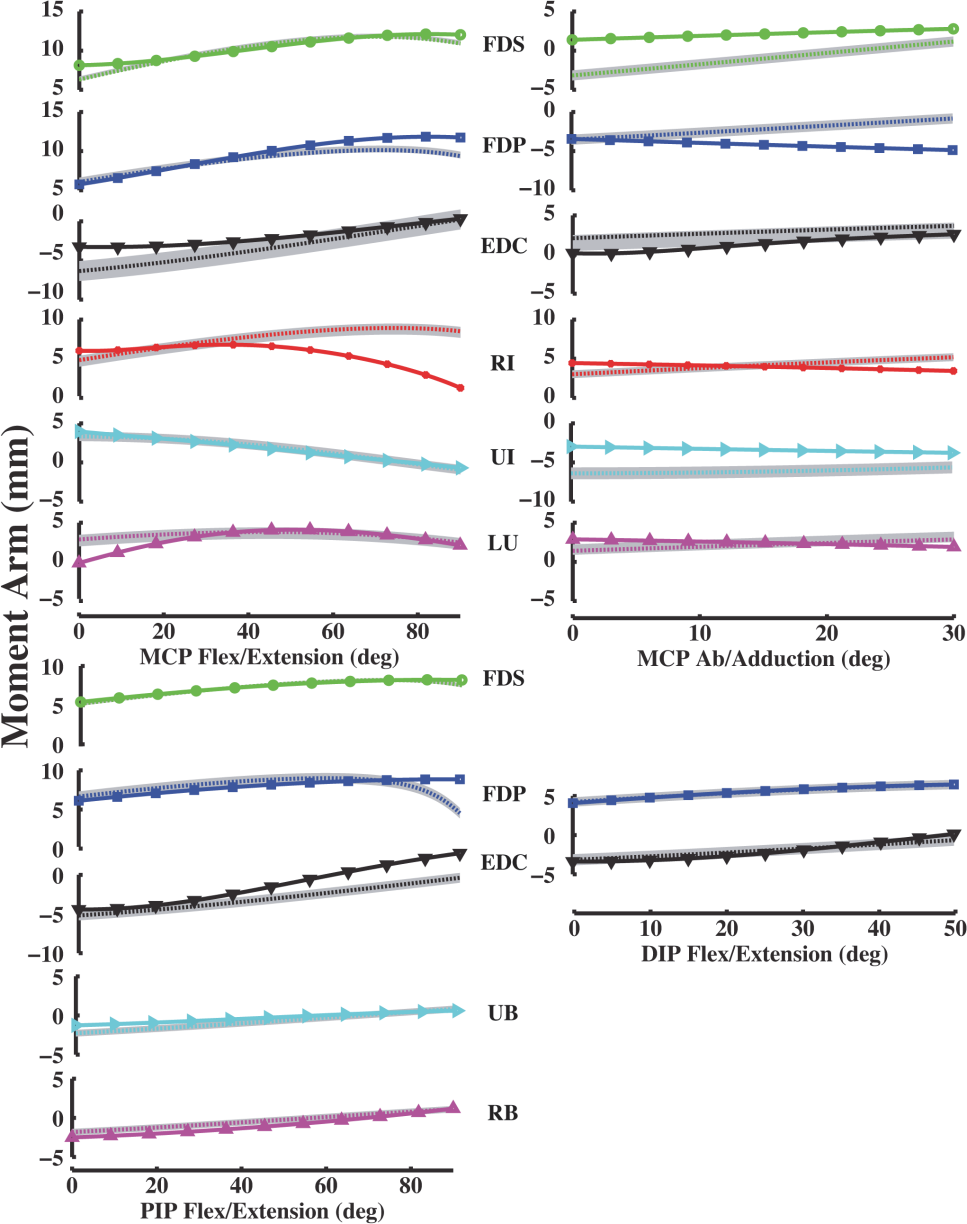
Little finger moment arm values (mm). Left MCP curves, PIP and DIP curves represent flex/extension moment arms as a function of flexion (+)/ extension (-). Right MCP curves represent ab/adduction moment arms as a function of abduction (+)/ adduction (-). Solid curves (with plot markers) represent experimentally derived moment arms from anatomical attachment locations, and dotted curves represent optimally estimated moment arms from data-driven optimizations.

Model-optimized moment arms agreed with experimentally derived values for the index, middle, ring and little fingers at the MCP joint (Figs 6–9). VAF averaged 84.3, 80.4, 86.6 and 84.1% for the index, middle, ring and little fingers, respectively (Table 3, 4, 5, 6). At the middle finger (min. VAF = 80.4%), RMS error ranged from min. 0.3 mm (UI) to max. 2.4 mm (EDC). Because S.D of moment arms has not been reported for any finger but the index finger, we compared the RMS errors to the S.D of index finger moment arms. Max. RMS error between computationally optimized and experimentally derived moment arm values was within one S.D of the experimentally measured index finger moment arm for all muscles (mean RMS error = 1.5 mm < measured S.D = 2.5 mm; [20]).

**Table 3:** Mean moment arms (MA) of the index finger.

Joint	RoM		FDP	FDS	EDC(ES)	LU(RB)	RI	UI(UB)
**MCP2**		**MA**	11.4	12.4	-7.4	3.8	5.5	1.9
	**0°~90°**	**(VAF)**	(98.9%)	(99.2%)	(91.2%)	(73.0%)	(57.4%)	(86.1%)
	**Flex/Ext**	**RMS**	Δ0.1	Δ0.1	Δ0.7	Δ1.0	Δ2.4	Δ0.3
		**(Error)**	(1.0%)	(0.7%)	(7.7%)	(19.8%)	(42.0%)	(28.6%)
		**MA**	1.3	0.7	-1.0	6.5	5.6	-6.7
	**0°~30°**	**(VAF)**	(5.3%)	(12.7%)	(89.9%)	(66.5%)	(76.6%)	(88.9%)
	**Ab/Add**	**RMS**	Δ1.7	Δ0.9	Δ0.1	Δ2.2	Δ1.3	Δ0.7
		**(Error)**	(59.5%)	(53.2%)	(8.6%)	(50.4%)	(31.7%)	(9.3%)
**PIP2**		**MA**	8.5	7.4	-3.6	-2.6		0.6
	**0°~90°**	**(VAF)**	(97.7%)	(89.5%)	(44.5%)	(33.5%)		(71.8%)
	**Flex/Ext**	**RMS**	Δ0.2	Δ0.8	Δ2.0	Δ2.2		Δ0.3
		**(Error)**	(0.5%)	(9.8%)	(32.8%)	(14.3%)		(10.8%)
**DIP2**		**MA**	5.0		-3.1			
	**0°~50°**	**(VAF)**	(88.9%)		(58.2%)			
	**Flex/Ext**	**RMS**	Δ0.6		Δ1.3			
		**(Error)**	(10.7%)		(37.4%)			

All are expressed in millimeters (mm) in OpenSim: flexion(+)/extension(-) and abduction(+)/adduction(-) for the MCP, PIP and DIP joints. (VAF) represents Variance Accounted For, RMS represents root mean square error (Δ) between OpenSim model and experimentally measured values [20], and (Error) represents percentage error, respectively.

**Table 4:** Mean moment arms (MA) of the middle finger.

Joint	RoM		FDP	FDS	EDC(ES)	LU(RB)	RI	UI(UB)
**MCP3**		**MA**	11.8	15.7	-6.7	4.0	8.0	2.2
	**0°~90°**	**(VAF)**	(83.6%)	(88.7%)	(65.3%)	(75.3%)	(81.5%)	(88.0%)
	**Flex/Ext**	**RMS**	Δ2.0	Δ1.8	Δ2.4	Δ1.0	Δ1.5	Δ0.3
		**(Error)**	(10.8%)	(6.9%)	(23.1%)	(23.0%)	(24.7%)	(6.2%)
		**MA**	-0.1	0.3	0.4	3.9	8.1	-3.8
	**0°~30°**	**(VAF)**	(8.9%)	(4.9%)	(29.8%)	(61.6%)	(63.2%)	(28.8%)
	**Ab/Add**	**RMS**	Δ2.6	Δ2.0	Δ0.6	Δ1.5	Δ3.0	Δ2.7
		**(Error)**	(84.8%)	(36.7%)	(51.6%)	(54.2%)	(56.4%)	(69.4%)
**PIP3**		**MA**	7.9	6.5	-4.2	-1.1		-0.2
	**0°~90°**	**(VAF)**	(94.5%)	(98.8%)	(42.6%)	(87.3%)		(86.0%)
	**Flex/Ext**	**RMS**	Δ0.4	Δ0.1	Δ2.4	Δ0.1		Δ0.3
		**(Error)**	(3.2%)	(0.7%)	(52.5%)	(1.1%)		(67.3%)
**DIP3**		**MA**	5.2		-2.8			
	**0°~50°**	**(VAF)**	(90.6%)		(72.2%)			
	**Flex/Ext**	**RMS**	Δ0.5		Δ0.8			
		**(Error)**	(9.7%)		(22.6%)			

All are expressed in millimeters (mm) in OpenSim: flexion(+)/extension(-) and abduction(+)/adduction(-) for the MCP, PIP and DIP joints. (VAF) represents Variance Accounted For, RMS represents root mean square error (Δ) between OpenSim model and experimentally measured values [20], and (Error) represents percentage error, respectively.

**Table 5:** Mean moment arms (MA) of the ring finger.

Joint	RoM		FDP	FDS	EDC(ES)	LU(RB)	RI	UI(UB)
**MCP4**		**MA**	9.1	9.7	-5.8	3.0	5.4	1.7
	**0**°**~90**°	**(VAF)**	(83.0%)	(87.2%)	(93.3%)	(75.3%)	(91.5%)	(89.1%)
	**Flex/Ext**	**RMS**	Δ1.6	Δ1.3	Δ0.4	Δ0.7	Δ0.5	Δ0.2
		**(Error)**	(9.6%)	(11.3%)	(1.1%)	(1.9%)	(3.8%)	(2.9%)
		**MA**	-2.1	0.1	3.2	4.0	2.8	-3.8
	**0**°**~30**°	**(VAF)**	(30.0%)	(84.1%)	(27.3%)	(1.6%)	(65.6%)	(53.4%)
	**Ab/Add**	**RMS**	Δ1.6	Δ1.6	Δ2.4	Δ4.0	Δ1.0	Δ1.8
		**(Error)**	(38.8%)	(75.1%)	(71.8%)	(96.8%)	(5.1%)	(60.2%)
**PIP4**		**MA**	6.2	5.0	-2.9	-0.4		-0.7
	**0**°**~90**°	**(VAF)**	(95.5%)	(95.0%)	(93.4%)	(84.1%)		(93.2%)
	**Flex/Ext**	**RMS**	Δ0.3	Δ0.3	Δ1.4	Δ0.1		Δ0.0
		**(Error)**	(2.0%)	(4.6%)	(48.2%)	(16.3%)		(6.2%)
**DIP4**		**MA**	3.3		-1.7			
	**0**°**~50**°	**(VAF)**	(96.4%)		(77.8%)			
	**Flex/Ext**	**RMS**	Δ0.1		Δ0.4			
		**(Error)**	(3.5%)		(3.0%)			

All are expressed in millimeters (mm) in OpenSim: flexion(+)/extension(-) and abduction(+)/adduction(-) for the MCP, PIP and DIP joints. (VAF) represents Variance Accounted For, RMS represents root mean square error (Δ) between OpenSim model and experimentally measured values [20], and (Error) represents percentage error, respectively.

**Table 6:** Mean moment arms (MA) of the little finger.

Joint	RoM		FDP	FDS	EDC(ES)	LU(RB)	RI	UI(UB)
**MCP5**		**MA**	8.8	10.4	-2.2	3.4	6.2	1.7
	**0°~90°**	**(VAF)**	(87.8%)	(96.7%)	(78.2%)	(70.5%)	(81.8%)	(89.6%)
	**Flex/Ext**	**RMS**	Δ1.1	Δ0.3	Δ0.6	Δ1.0	Δ1.2	Δ0.2
		**(Error)**	(8.9%)	(0.1%)	(13.1%)	(14.1%)	(11.7%)	(1.3%)
		**MA**	-2.2	2.2	2.9	2.4	4.1	-6.1
	**0°~30°**	**(VAF)**	(99.2%)	(96.7%)	(73.5%)	(69.3%)	(77.5%)	(54.7%)
	**Ab/Add**	**RMS**	Δ2.3	Δ5.3	Δ1.6	Δ0.8	Δ0.9	Δ2.8
		**(Error)**	(31.5%)	(91.5%)	(74.9%)	(29.1%)	(4.3%)	(57.6%)
**PIP5**		**MA**	8.0	7.2	-3.0	-0.4		-0.8
	**0°~90°**	**(VAF)**	(86.7%)	(97.7%)	(42.8%)	(33.7%)		(52.1%)
	**Flex/Ext**	**RMS**	Δ1.1	Δ0.2	Δ1.9	Δ0.6		Δ0.6
		**(Error)**	(9.8%)	(1.7%)	(76.1%)	(34.5%)		(86.6%)
**DIP5**		**MA**	5.5		-2.0			
	**0°~50°**	**(VAF)**	(99.1%)		(80.1%)			
	**Flex/Ext**	**RMS**	Δ0.1		Δ0.4			
		**(Error)**	(0.8%)		(24.7%)			

All are expressed in millimeters (mm) in OpenSim: flexion(+)/extension(-) and abduction(+)/adduction(-) for the MCP, PIP and DIP joints. (VAF) represents Variance Accounted For, RMS represents root mean square error (Δ) between OpenSim model and experimentally measured values [20], and (Error) represents percentage error, respectively.

For the PIP and DIP joints, model-optimized moment arms also matched experimentally derived data (Figs 6–9). VAF averaged 67.4, 81.8, 92.2 and 62.6% for muscles at the index, middle, ring and little finger PIP joints, respectively. For the DIP joints, VAF averaged 73.5, 81.4, 87.1 and 89.6% across all muscles for the index, middle, ring and little fingers, respectively. RMS error between computationally optimized and experimentally derived moment arm values was within one S.D of the experimentally measured index finger moment arm for all muscles (mean RMS error = 0.7 mm < measured S.D = 1.3 mm; [20]).

## Discussion

The goal of this study was to determine attachment points for muscles of the index, middle, ring and little fingers in the OpenSim upper-extremity model. Moment arms calculated from experimentally measured muscle attachments using the partial velocity method matched experimentally measured moment arms for the index finger, where both attachments and moment arm data were available. Optimization found OpenSim muscle attachments resulting in moment arms that matched moment arms derived from experimental measurements.

### Limitations

Our approach had several limitations. First, the muscle wrapping objects that we used during optimization were simple shapes that in some circumstances can result in discontinuous moment arm curves [9]. Muscle wrapping can be used to better describe muscle-tendon geometry [44,45]. However, to our knowledge quantitative, experimental data for wrapping surfaces are not available for finger muscles. Therefore, we chose to use minimal wrapping surfaces that prevented the muscle-tendon paths from penetrating the bone, but did not otherwise change the muscle-tendon paths.

Second, compiling data drawn from different reference frames and sources into a common model could introduce errors. We attempted to mitigate these potential sources of error by primarily basing our calculations on data collected from all fingers from the same specimens [5,20]. Moreover, we normalized not only by middle phalanx length for flex/extension moment arms at the PIP and DIP joints, but also normalized by MCP thickness for flex/extension moment arms and MCP width for ab/adduction values.

Third, although it was possible to estimate experimentally derived muscle attachment locations in OpenSim, the coordinate transformations involved several assumptions or approximations. Attachment estimations required both scaling and coordinate transformations from 2D to 3D. Coordinate transformations are complicated by the absence of quantitative information for both 3D rotation and translation in the 2D model. We were therefore required to make assumptions about both 3D reference frame orientations and origins. However, the discovery of muscle attachments that were similar to finger anatomy suggests that our assumptions were reasonable (i.e., low ΔR = 4.9 mm, which was less than the 6.9 mm interspecimen phalanx variation observed experimentally; Tables 7–10). Moreover, optimization resulted in muscle moment arms that matched experimentally measured values. Therefore, even though the attachment sites are for a hypothetical 50^th^ percentile male and not for a specific specimen, muscle function is as close to experimentally measured values as feasible.

**Table 7:** Index finger muscle-tendon locations, expressed in OpenSim frame (mm).

Joint	Muscle	x	y	z	ΔR	x	y	z	ΔR
		**proximal point (secondmc)**				**distal point (proxph2)**			
	**FDP**	4.11	-16.00	-4.21	5.6	3.34	-20.24	-5.03	9.0
		(2.76)	(-21.47)	(-4.02)		(1.00)	(-13.35)	(-9.54)	
	**FDS**	4.86	-13.77	-0.66	9.8	1.32	-8.41	-12.01	5.4
		(1.65)	(-21.47)	(-5.83)		(-0.83)	(-13.35)	(-12.12)	
	**RI**	9.07	-19.95	-4.88	4.1	7.04	-6.72	-0.15	11.7
		(10.45)	(-19.21)	(-1.11)		(7.20)	(-18.37)	(0.50)	
**MCP2**	**LU**	10.17	-26.47	-0.01	10.2	8.38	-8.29	0.04	10.9
		(10.63)	(-19.21)	(-7.10)		(6.69)	(-18.37)	(-3.66)	
	**UI**	-3.32	-29.39	-0.12	10.2	-4.31	-15.93	2.41	5.5
		(-4.36)	(-19.21)	(-0.39)		(-6.96)	(-18.37)	(-1.68)	
	**EDC**	3.05	-29.51	12.43	2.9	3.31	-7.11	11.64	4.8
	**(LE)**	(2.96)	(-28.24)	(15.05)		(0.33)	(-10.84)	(11.42)	
		**proximal point (proxph2)**				**distal point (midph2)**			
	**FDP**	6.33	-34.33	0.61	3.3	0.61	-8.81	-5.84	1.8
		(8.01)	(-36.25)	(9.35)		(0.68)	(-8.89)	(-7.66)	
	**RB**	11.30	-33.93	9.35	6.1	5.80	-6.86	8.79	3.3
	**(LU)**	(10.79)	(-40.02)	(9.86)		(5.20)	(-6.38)	(5.50)	
	**UB**	1.00	-39.44	11.14	3.3	-4.08	-6.55	3.75	3.5
**PIP2**	**(UI)**	(3.16)	(-40.02)	(8.78)		(-7.61)	(-6.38)	(3.74)	
	**FDS**	6.35	-34.33	1.61	3.0	-0.24	-8.81	-4.08	2.0
		(7.55)	(-36.25)	(3.64)		(0.13)	(-8.89)	(-6.02)	
	**ES**	6.88	-38.72	10.95	2.6	1.86	-0.10	7.41	5.3
	**(EDC)**	(7.02)	(-41.27)	(10.84)		(-0.90)	(-4.62)	(7.35)	
		**proximal point (midph2)**				**distal point (distph2)**			
	**TE**	2.83	-24.89	4.82	0.0	-0.48	-5.62	3.45	2.0
**DIP2**	**(EDC)**	(2.83)	(-24.89)	(4.82)		(-0.48)	(-5.62)	(5.45)	
	**FDP**	4.32	-19.62	-3.52		-0.70	-5.62	-3.99	0.0
		(4.32)	(-19.62)	(-3.52)		(-0.70)	(-5.62)	(-3.99)	

The coordinate system of the OpenSim model is attached to metacarpal (secondmc), proximal (proxph2), middle (midph2) and distal (distph2) phalanges. x, y and z components indicate radioulnar (+ points out, perpendicular to the palm plan), axial (+ points from distal to proximal side) and dorsolar (+ points up, from palm to hand side), respectively. Optimized muscle-tendon locations of OpenSim are compared with those of experimental measurements (numbers in parentheses). ΔR is the Euclidean distance between OpenSim model and experimentally measured attachment sites [5].

**Table 8:** Middle finger muscle-tendon locations, expressed in OpenSim frame (mm).

Joint	Muscle	x	y	z	ΔR	x	y	z	ΔR
		**proximal point (thirdmc)**				**distal point (proxph3)**			
	**FDP**	1.86	-15.09	-6.77	5.6	0.69	-15.83	-8.10	1.4
		(0.53)	(-19.14)	(-3.07)		(0.61)	(-14.58)	(-8.75)	
	**FDS**	2.24	-10.87	-13.45	11.5	2.44	-19.19	-8.01	6.2
		(0.69)	(-19.14)	(-5.55)		(-1.03)	(-14.58)	(-10.41)	
	**RI**	9.91	-24.00	-4.96	7.9	7.70	-19.91	0.05	2.7
		(6.88)	(-16.75)	(-5.34)		(5.26)	(-19.88)	(-1.01)	
**MCP3**	**LU**	10.25	-23.17	-0.83	9.6	6.84	-21.96	7.26	11.3
		(6.18)	(-16.75)	(-5.34)		(5.22)	(-19.88)	(-3.71)	
	**UI**	-3.11	-24.84	-3.45	13.8	-1.19	-18.29	-9.33	11.3
		(-4.98)	(-16.75)	(-14.43)		(-5.68)	(-19.88)	(0.95)	
	**EDC**	-0.69	-26.29	10.04	1.1	-1.18	-12.22	10.74	2.3
	**(LE)**	(-0.69)	(-26.29)	(11.15)		(-0.48)	(-11.93)	(8.53)	
		**proximal point (proxph3)**				**distal point (midph3)**			
	**FDP**	2.38	-36.43	0.54	3.4	1.58	-11.49	-5.12	2.9
		(1.60)	(-38.91)	(2.71)		(-0.11)	(-10.34)	(-7.23)	
	**RB**	5.79	-44.69	7.56	2.0	4.92	-11.61	3.54	4.5
	**(LU)**	(4.86)	(-42.89)	(7.56)		(6.20)	(-7.69)	(4.56)	
	**UB**	-4.26	-42.83	6.94	2.2	-5.36	-7.74	5.97	1.2
**PIP3**	**(UI)**	(-2.05)	(-42.89)	(7.24)		(-6.55)	(-7.69)	(4.27)	
	**FDS**	4.54	-37.60	2.13	3.6	0.23	-10.75	-5.41	4.6
		(1.44)	(-38.91)	(3.35)		(0.03)	(-10.34)	(-5.49)	
	**ES**	1.69	-41.39	11.27	3.4	0.39	-4.23	6.25	3.9
	**(EDC)**	(1.40)	(-44.21)	(9.36)		(-0.64)	(-3.71)	(6.55)	
		**proximal point (midph3)**				**distal point (distph3)**			
	**TE**	1.09	-29.05	5.07	0.0	-0.58	-6.94	3.08	1.5
**DIP3**	**(EDC)**	(1.09)	(-29.05)	(5.07)		(-0.58)	(-5.83)	(4.08)	
	**FDP**	2.13	-21.44	-2.13	0.0	0.06	-5.01	-4.19	2.0
		(1.98)	(-23.48)	(-2.76)		(-0.85)	(-3.18)	(-3.74)	

Parentheses () values indicate measured muscle-tendon locations [5], transformed to OpenSim coordinate. ΔR is the Euclidean distance between OpenSim model and experimentally measured attachment sites [5].

**Table 9:** Ring finger muscle-tendon locations, expressed in OpenSim frame (mm).

Joint	Muscle	X	y	z	ΔR	x	y	z	ΔR
		**proximal point (fourthmc)**				**distal point (proxph4)**			
	**FDP**	-0.62	-13.64	-6.29	4.1	-1.22	-11.97	-8.80	2.5
		(-2.57)	(-16.97)	(-7.76)		(1.00)	(-10.96)	(-9.46)	
	**FDS**	2.02	-13.83	-6.02	6.7	0.08	-12.68	-7.47	5.0
		(-3.67)	(-16.97)	(-9.55)		(-0.82)	(-10.96)	(-12.03)	
	**RI**	1.63	-17.90	-9.78	8.1	9.87	-16.29	0.53	2.8
		(7.18)	(-14.73)	(-4.86)		(7.14)	(-15.94)	(0.50)	
**MCP4**	**LU**	3.17	-22.42	-0.81	13.3	0.17	-14.60	0.62	7.9
		(7.36)	(-14.73)	(-10.80)		(6.63)	(-15.94)	(-3.64)	
	**UI**	-4.85	-24.09	-4.39	9.7	-4.15	-21.18	-5.52	9.3
		(-7.51)	(-14.73)	(-4.15)		(-6.90)	(-15.94)	(-1.67)	
	**EDC**	-0.42	-20.69	9.47	4.0	-1.99	-8.36	8.76	3.1
	**(LE)**	(-2.37)	(-23.69)	(11.17)		(-0.32)	(-8.47)	(11.33)	
		**proximal point (proxph4)**				**distal point (midph4)**			
	**FDP**	-3.44	-33.34	-1.92	2.4	2.32	-7.12	-6.00	2.4
		(-3.04)	(-35.28)	(-3.32)		(0.67)	(-7.84)	(-7.59)	
	**RB**	1.28	-40.33	4.10	2.1	4.27	-4.51	4.30	1.6
	**(LU)**	(-0.28)	(-39.01)	(3.86)		(5.15)	(-5.35)	(5.45)	
	**UB**	-8.87	-38.84	1.83	1.6	-5.76	-4.01	3.39	2.3
**PIP4**	**(UI)**	(-7.85)	(-39.01)	(2.79)		(-7.55)	(-5.35)	(3.71)	
	**FDS**	0.56	-36.10	-0.99	4.3	0.04	-11.94	-1.77	5.9
		(-3.49)	(-35.28)	(2.32)		(0.12)	(-7.84)	(-5.98)	
	**ES**	-3.85	-38.00	4.84	2.5	-1.94	-5.82	8.82	3.5
	**(EDC)**	(-4.03)	(-40.26)	(4.83)		(-0.90)	(-3.61)	(7.30)	
		**proximal point (midph4)**				**distal point (distph4)**			
	**TE**	0.49	-23.94	3.56	3.3	-0.39	-2.72	3.58	1.0
**DIP4**	**(EDC)**	(-2.69)	(-24.79)	(4.14)		(-0.47)	(-3.74)	(5.40)	
	**FDP**	-0.70	-19.70	-1.31	2.9	0.33	-4.04	-3.05	1.4
		(-1.34)	(-19.56)	(-4.14)		(0.70)	(-3.74)	(-3.96)	

Parentheses () values indicate measured muscle-tendon locations [5], transformed to OpenSim coordinate. ΔR is the Euclidean distance between OpenSim model and experimentally measured attachment sites [5].

**Table 10:** Little finger muscle-tendon locations, expressed in OpenSim frame (mm).

Joint	Muscle	x	y	z	ΔR	x	y	z	ΔR
		**proximal point (fifthmc)**				**distal point (proxph5)**			
	**FDP**	0.95	-13.05	-8.70	4.3	-0.95	-13.66	-5.11	3.6
		(-2.22)	(-15.76)	(-7.89)		(0.84)	(-12.39)	(-7.98)	
	**FDS**	-0.10	-13.08	-9.52	4.1	-1.67	-14.93	-8.12	3.4
		(-3.15)	(-15.76)	(-9.40)		(-0.69)	(-12.39)	(-10.14)	
	**RI**	6.11	-14.87	-6.60	1.5	4.72	-14.34	2.33	3.2
		(6.03)	(-13.87)	(-5.45)		(6.02)	(-16.59)	(0.42)	
**MCP5**	**LU**	6.06	-19.91	-0.17	11.7	3.51	-13.13	1.96	5.2
		(6.17)	(-13.87)	(-10.46)		(5.59)	(-16.59)	(-3.07)	
	**UI**	-5.13	-19.67	-7.63	6.5	-3.07	-17.71	-7.93	7.2
		(-6.37)	(-13.87)	(-4.85)		(-5.82)	(-16.59)	(-1.41)	
	**EDC**	-1.26	-18.18	5.66	4.1	-4.45	-12.53	6.29	5.8
	**(LE)**	(-2.05)	(-21.43)	(8.07)		(-0.27)	(-10.29)	(9.56)	
		**proximal point (proxph5)**				**distal point (midph5)**			
	**FDP**	-0.46	-29.14	-8.05	5.1	0.87	-8.31	-7.69	1.6
		(-4.87)	(-31.36)	(-6.82)		(0.57)	(-7.46)	(-6.41)	
	**RB**	-3.16	-37.20	3.14	4.9	1.39	-7.41	2.74	4.1
	**(LU)**	(-2.54)	(-34.51)	(-0.76)		(4.35)	(-5.36)	(4.60)	
	**UB**	-10.71	-35.88	-3.16	2.7	-3.89	-7.41	2.74	7.2
**PIP5**	**(UI)**	(-8.92)	(-34.51)	(-1.66)		(-6.36)	(-5.36)	(3.13)	
	**FDS**	-2.51	-25.87	-5.41	6.2	2.11	-5.50	-6.96	3.4
		(-5.25)	(-31.36)	(-5.97)		(0.11)	(-7.46)	(-5.04)	
	**ES**	-7.94	-32.24	2.51	4.7	-2.68	-7.36	4.66	4.2
	**(EDC)**	(-5.70)	(-35.56)	(0.06)		(-0.76)	(-3.89)	(6.15)	
		**proximal point (midph5)**				**distal point (distph5)**			
	**TE**	0.20	-20.00	6.42	6.6	1.48	-13.95	4.70	9.7
**DIP5**	**(EDC)**	(-3.17)	(-20.68)	(0.77)		(-0.39)	(-4.41)	(4.56)	
	**FDP**	-1.01	-16.01	-4.87	3.3	0.19	-11.35	-4.68	7.1
		(-2.05)	(-16.27)	(-6.21)		(0.59)	(-4.41)	(-3.34)	

Parentheses () values indicate measured muscle-tendon locations [5], transformed to OpenSim coordinate. ΔR is the Euclidean distance between OpenSim model and experimentally measured attachment sites [5].

Finally, we did not conduct experiments to validate the model at a functional level [13]. Full model validation was outside of the scope of the present study because it would require the determination of muscle physiological parameters and measurements of muscle activation during diverse tasks. Moreover, modeling potentially complex structures such as the extensor mechanism may require additional data, assumptions, and validation [46]. We therefore limited model validation to the anatomical level of muscle attachment locations. Future experiments will seek to functionally validate the model and resolve discrepancies among experimental measurements [20,21,40].

### Moment Arms and Muscle Attachments were Reasonable

Moment arms calculated using the partial velocity method for the middle, ring and little finger muscles (whose experimentally measured moment arms are not available) were reasonable based on moment arm magnitude and variation with joint angle. Flex/extension moment arms had the same ordering of magnitude as the finger dimensions, e.g., moment arms for the middle finger largest, ring or index second, and little smallest [40]. Joint thickness or phalanx lengths influenced moment arms, e.g., moment arm magnitudes increased with phalanx lengths [20,21,35,40]. Moreover, calculated moment arm variation with joint angle was consistent among muscles at all finger joints. For example, calculated flexor moment arms increased with finger flexion; calculated extensor values decreased. Experimentally measured moment arms for extrinsic (FDP/FDS) and intrinsic (RI/UI/LU) flexors increased with flexion for the index, middle, ring and little fingers, whereas extensors (TE/ES/EDC) increased with extension as observed in previous studies [5,6,20,23,24,36]. Therefore, the partial velocity method predicted moment arm values whose relationships to joint angle agreed with experimental data.

Muscle attachments found by optimization were also reasonable. Optimization identified multiple muscle attachments that locally minimized RMS error (Fig 5). However, at least one set of muscle attachments were close to experimentally measured attachments. The average distance between modeled and measured muscle attachments fell within 7.1% of the average length of the muscle paths and 71.0% of one standard deviation of specimens’ phalanx length. Moreover, the agreement of the OpenSim moment arms with both directly measured moment arms [20] and moment arms calculated from muscle attachments [5] suggested that the OpenSim muscle attachments are reasonable within the OpenSim coordinate system.

Although optimization found moment arm curves that closely matched experimental values for most muscles, some muscles such as UI showed poorer fits. Differences in VAF may in part be related to variability in experimentally measured data. An et al. (1983) reported more variability in ab/adduction moment arm (S.D = 2.5 mm) than that of flex/extension (S.D = 2.1 mm). Ab/adduction moment arms could also be influenced by finger postures to a greater extent than flex/extension moment arms [41]. Moreover, although RMS error was the same for ab/adduction and flex/extension, VAF was lower for ab/adduction because of smaller overall moment arms than flex/extension. Ab/adduction moment arms (mean MA = 3.4 mm) were less than half the magnitude of flex/extension values (mean MA = 7.2 mm). Therefore, low VAF values for some muscles may reflect variability, experimental uncertainty, and small overall moment arm values.

### Objective Methods could Facilitate Transformation among Musculoskeletal Models

The optimization procedure we proposed could potentially help to translate data from one musculoskeletal model to another. We demonstrated that reasonable muscle attachments can be determined using muscle function (e.g., moment arms) as a constraint. Scaling or individualizing musculoskeletal models can be difficult. Often, scaling is based on body segment lengths or limb circumferences. For example, scaling has been based on finger middle phalanx length [5], scaling elbow moment arm by cross-sectional area [47], or other linear dimensions [48]. However, scaling alone does not account for other differences among models such as different axes of rotation [34]. Using both scaling and data-driven optimization of functional parameters can identify muscle-tendon paths that both maintain anatomical proportionality and muscle function at the same time.

### Muscle Parameters for a Dynamic Model

We added only anatomical information to the OpenSim model. Musculoskeletal anatomy alone can be helpful for understanding some aspects of function such as joint stability [49]. However, to dynamically simulate movement, modeling additional aspects of muscle anatomy, physiology, and motor control will be necessary. Muscle anatomical properties can be drawn from literature values, including physiological cross-sectional area and optimal fiber length [22], pennation angle [50–53], and tendon slack length [54,55]. Likewise, physiological properties such as passive and active force-length, series elasticity, force-velocity, force-activation, maximum isometric muscle force, and optimal muscle fiber length can be modeled based on experimental measurements [9,56,57]. Muscle activity from experimental measurements can be used to predict joint loading and to better understand joint function during specific tasks [58,59]. The muscle attachments we report will allow for dynamic models of tasks involving many fingers or the entire hand.

Addition of finger muscles to the OpenSim upper-extremity model will facilitate efforts to use the model to better characterize dexterous finger movements. Coupled with experimental data, the model will allow estimates of internal musculoskeletal loading during multi-touch tasks involving many fingers. The model will therefore aid understanding of complex multi-touch and gestural movements, and potentially guide the design of technologies that reduce injury risk.
