# Correction: Finger Muscle Attachments for an OpenSim Upper-Extremity Model

**DOI:** 10.1371/journal.pone.0267620

**Published:** 2022-04-20

**Authors:** Jong Hwa Lee, Deanna S. Asakawa, Jack T. Dennerlein, Devin L. Jindrich

In the Musculoskeletal Model subsection of the Methods section, the first sentence of the second paragraph contains incorrect, outdated terminology when referring to the palmar interosseous and dorsal interosseous muscles. The correct sentence is:

“For all fingers, we modeled the following muscles: *terminal extensor (TE)*, *extensor slip (ES)*, *radial band (RB)*, *ulnar band (UB)*, *radial interosseous (RI)*, *lumbricals (LU)*, *ulnar interosseous (UI)*, *flexor digitorum profundus (FDP)*, *flexor digitorum superficialis (FDS) and extensor digitorum communis or long extensor (EDC)*.”
